# ﻿A new subspecies of *Stellariaalsine* (Caryophyllaceae) from Yakushima, Japan

**DOI:** 10.3897/phytokeys.187.64023

**Published:** 2021-12-30

**Authors:** Tetsukazu Yahara, Shun K. Hirota, Kengo Fuse, Hiroyuki Sato, Shuichiro Tagane, Yoshihisa Suyama

**Affiliations:** 1 Kyushu Open University, 744 Motooka, Fukuoka, 819-0395, Japan Kyushu Open University Fukuoka Japan; 2 Field Science Center, Graduate School of Agricultural Science, Tohoku University, 232-3 Aza-yomogida, Naruko Onsen, Osaki, Miyagi 989-6711, Japan Tohoku University Osaki Japan; 3 The Kagoshima University Museum, Kagoshima University, 1-21-30 Korimoto, Kagoshima, 890-0065, Japan Kagoshima University Fukuoka Japan

**Keywords:** cpDNA, DNA barcoding, island, ITS, MIG-seq, next generation sequencing, threatened plants

## Abstract

An unknown taxon of *Stellaria* was discovered in Yakushima, a Japanese island known to harbor several endemic species. To determine the identity of this taxon, this study employed MIG-seq for the reconstruction of a finely resolved phylogenetic tree of the newly discovered taxon, along with some related species of *Stellaria*. The results showed that the newly discovered taxon is a relative of *S.alsine*. Based on this result, Stellariaalsinesubsp.nana**subsp. nov.** was published.

## ﻿Introduction

*Stellaria* L. is a diverse genus belonging to the family Caryophyllaceae (tribe Arenarieae), and comprises approximately 190 species distributed primarily in the temperate areas of the Northern Hemisphere (Chen and Rabeler 2001). In Japan, 19 species have been recorded to date by Kadota (2017), including a recently discovered species, *S.hibinoi*, as described by Serizawa (2015).

This study describes an additional new taxon that inhabits the mountainous area of Yakushima, an island where a variety of endemic plant taxa have been previously recorded (Yahara et al. 1987). This taxon has a dwarf stem, 2.5–6 cm long. Morphologically, this taxon is similar to *S.alsine* Grimm, but it is difficult to determine the taxonomic relationship of this dwarf plant based solely on morphological observations. Thus, we carried out a phylogenetic analysis of *S.alsine* and the newly discovered taxon, as well as several other species of *Stellaria* using MIG-seq (multiplexed ISSR genotyping by sequencing; Suyama and Matsuki 2015). MIG-seq is capable of efficiently detecting genome-wide SNPs using inter-simple sequence repeats (ISSRs) as multiplex PCR primers. This method has been applied successfully to taxonomically differentiate between groups that are difficult to classify, such as Fagaceae (Binh et al. 2018; Strijk et al. 2020), Lauraceae (Zhang et al. 2020) and Asparagaceae (Yahara et al. 2021), in order to reconstruct highly resolved phylogenetic relationships among closely related species and infraspecific taxa. The MIG-seq tree obtained in this study demonstrates that the newly discovered taxon is sister to, but highly diverged from, *S.alsine*. Based on this finding, we provide a formal description of S.alsinesubsp.nana subsp. nov. and discuss the implications of this discovery.

## ﻿Methods

### ﻿Field survey

Yakushima (Yaku Island) is a roughly circular island with a circumference of approximately 130 km and is located approximately 60 km south of the main island of Kyushu. Since the initial taxonomic review of 45 species of vascular plants endemic to Yakushima (Yahara et al. 1987), six additional new species have been described in the same region (Yahara and Tsukaya 2008; Katsuyama 2009; Chen et al. 2014; Hori et al. 2015; Suetsugu and Fukunaga 2016; Suetsugu et al. 2016). Furthermore, an unknown taxon of *Cardamine* was also discovered in the mountainous area of Yakushima (Kudoh 2017). Between July 19 and July 24, 2020, a field trip was made to the mountainous area of Yakushima in order to collect this unknown species of *Cardamine*, whereby we serendipitously discovered a plant with cleistogamous flowers of the unknown taxon of *Stellaria*. Later, fruiting specimens of this taxon were collected on September 4, 2020, and a specimen with a chasmogamous flower was collected on May 3, 2021 in the same area by K. Fuse. In addition, we collected samples of S.alsinesubsp.alsine, *S.aquatica* (L.) Scop., *S.diversiflora* Maxim., *S.media* (L.) Vill., *S.monosperma* Buch.-Ham. ex D. Don, and *S.neglecta* Weihe (Table 1), and examined phylogenetic relationships.

**Table 1.** A list of samples used in the molecular phylogenetic analyses.

Scientific name	Voucher ID	Locality	Latitude, Longitude
Stellariaalsinesubsp.alsine	JPN0029	Itoshima, Fukuoka	33.48162500, 130.2413472
Stellariaalsinesubsp.alsine	JPN3306	Itoshima, Fukuoka	33.57076666, 130.2034750
Stellariaalsinesubsp.nana	JPN0573	Yakushima, Kagoshima	30.34137777, 130.4770056
Stellariaalsinesubsp.nana	JPN1791	Yakushima, Kagoshima	30.34171944, 130.4753583
*Stellariaaquatica*	JPN3046	Mitsukaido, Ibaraki	36.01760555, 139.9974056
*Stellariaaquatica*	JPN3106	Itoshima, Fukuoka	33.49042500, 130.2582250
*Stellariadiversiflora*	JPN1352	Mt. Yokogura, Kochi	33.53525555, 133.2013083
*Stellariamedia*	JPN0008	Itoshima, Fukuoka	33.48307500, 130.2636556
*Stellariamedia*	JPN0069	Itoshima, Fukuoka	33.47711944, 130.2409528
*Stellariamedia*	JPN0816	Itoshima, Fukuoka	33.47456666, 130.2512972
*Stellariamonosperma*	JPN2119	Mt. Shiraiwa, Miyazaki	32.57839166, 131.1143333
*Stellariamonosperma*	JPN2998	Osugi-dani, Mie	34.21359722, 136.1698139
*Stellarianeglecta*	JPN0006	Itoshima, Fukuoka	33.48307500, 130.2636556

### ﻿DNA isolation, sequencing, and construction of SNP-based phylogenetic trees

Total DNA was extracted from dried leaves using the CTAB method (Doyle and Doyle 1990). *De novo* SNP discovery was performed using MIG-seq (Suyama and Matsuki 2015). Based on the methodology described by Suyama and Matsuki (2015), a MIG-seq library was prepared via a two-step PCR amplification process with minor modifications, namely the annealing temperature of the first PCR was altered from 48 °C to 38 °C. Subsequently, the second PCR products were purified in the size range of 300–800 bp and sequenced on an Illumina MiSeq platform (Illumina, San Diego, CA, USA) using a MiSeq Reagent Kit v3 (150 cycle, Illumina). Sequencing of the first 17 bases of reads 1 and 2 (SSR primer regions and anchors) was bypassed using the ‘DarkCycle’ function of the MiSeq platform. Additionally, low-quality reads and extremely short reads containing adapter sequences were removed using the Trimmomatic 0.39 software (Bolger et al. 2014). The Stacks 2.41 pipeline (Catchen et al. 2013; Rochette et al. 2019) was used to obtain individual genotypes with the following parameters: minimum depth of coverage required to create a stack (*m*) = 3, maximum distance between stacks (*M*) = 2, and maximum mismatches between loci when building the catalog (*n*) = 2. Three different filtering criteria were applied for quality control of the SNP data. First, any SNP site where one of two alleles had less than three counts was filtered out due to the difficulty in distinguishing polymorphisms from sequencing errors that arise when the minor allele count of SNPs is too low (Roesti et al. 2012). Second, loci containing SNPs with high heterozygosity (*H*o ≥ 0.6) were removed as the excess heterozygosity may have resulted from artifactual loci constructed from several paralogous genomic regions. Third, the SNPs retained by three or more samples were included in the SNP dataset.

Maximum likelihood phylogeny based on SNPs was inferred using the RAxML 8.2.10 software (Stamatakis 2014). A GTRCAT model was applied during this process and 1,000 replicates of parallel tree search bootstrapping were performed.

### ﻿Phylogenetic analysis using chloroplast and nuclear genomic sequences

Three chloroplast and two nuclear genomic regions were sequenced using next-generation DNA sequencing. In this regard, *rbc*L, *trn*L intron, *psb*A-*trn*H, ITS1, and ITS2 were initially simultaneously amplified using the Multiplex PCR Assay Kit Ver. 2 (Takara Bio, Kusatsu, Japan) (first PCR). The first set of primers consisted of tail sequences and locus-specific primers (Suyama et al. 2022). Subsequently, the first PCR products were purified and used for the second PCR. The second PCR was conducted using primer pairs, including tail sequences, adapter sequences for Illumina sequencing, and the index sequence to identify each individual sample. In this step, the second PCR product from each sample was mixed and sequenced on the Illumina MiSeq platform using a MiSeq Reagent Nano Kit v2 (500 cycle, Illumina). The sequencing of the first three bases of reads 1 and 2 (anchor region for the 2^nd^ PCR primer) was bypassed using the ‘DarkCycle’ option of the MiSeq system. Both ends of the fragments and index sequences were read by paired-end sequences (reads 1 and 2) and index sequencing. The number of bases per read was 251 bases for read 1 and 251 bases for read 2.

The sequences of the five regions were determined using the Claident pipeline (Tanabe and Toju 2013, http://www.claident.org/, Tanabe, A.S., Claident, Date of access: 05/01/2021). The raw MiSeq BCL data were first converted into FASTQ data using the BCL2FASTQ program provided by Illumina, followed by demultiplexing of the raw FASTQ data based on index and primer sequences, using the clsplitseq program in Claident. Subsequent analysis of the data was performed per region per individual. In ITS1 and ITS2, the pair-end reads were merged since reads 1 and 2 were overlapping. In *rbc*L, *trn*L intron, and *psb*A-*trn*H, reads 1 and 2 were independently analyzed because the length of the sequenced reads was too short to allow for merging. Additionally, the low-quality 3' tail was trimmed, and the low-quality sequences were filtered out using the clfilterseq program. The noisy sequences were removed using the clcleanseqv program. Finally, the remaining reads were clustered with a cut-off sequence similarity of 99%. An OTU that had the most observed reads within the individual was treated as a representative OTU sequence.

Multiple alignments were performed using the MAFFT 7.313 program (Katoh and Standley 2013), and alignment columns containing gaps were trimmed using a heuristic selection method based on the similarity statistics of trimAl 1.4.rev15 (Capella-Gutierrez et al. 2009). The Kakusan 4.0 software (Tanabe 2011) was used to find suitable nucleotide substitution models and partitioning strategies for the nucleotide datasets by independently running the chloroplast and nuclear genomic regions through this program. The AICc criterion (Sugiura 1978) was used to compare the Nonpartitioned, the Partitioned_Equal_Mean_Rate, as well as the Separate models. For chloroplast genomic regions, the Partitioned_Equal_Mean_Rate model (GTR + Γ), which assumes an equal rate of nucleotide substitutions across arbitrarily specified partitions, proved optimal. Contrastingly, for nuclear genomic regions, the Nonpartitioned model (GTR + Γ) proved optimal. Maximum likelihood phylogenies were further inferred using RAxML 8.2.10 (Stamatakis 2014), whereby 1,000 replicates of parallel tree search bootstrapping were conducted.

## ﻿Data resources

All raw MIG-seq data were deposited at the DDBJ Sequence Read Archive (DRA) with accession number DRA011466. The demultiplexed raw reads of ITS and cpDNA regions were deposited at the DDBJ Sequence Read Archive (DRA) and assigned Accession no. DRA011467.

## ﻿Results

### ﻿MIG-seq tree

A total of 15,551,282 raw reads (1,196,252 ± 87,288 reads per sample) were obtained via MIG-seq, of which 14,923,278 reads (1,147,944 ± 84,758 reads per sample) remained after quality control. Following the *de novo* SNP detection and filtering, a dataset comprised of 881 SNPs from 703 loci was obtained. Three samples – Stmon-JPN2119, Stmon-JPN2998, and Stdiv-JPN1352 – contained more than 90% of the miscalled SNPs; thus, these samples were eliminated from further SNP analysis.

**Figure 1.**
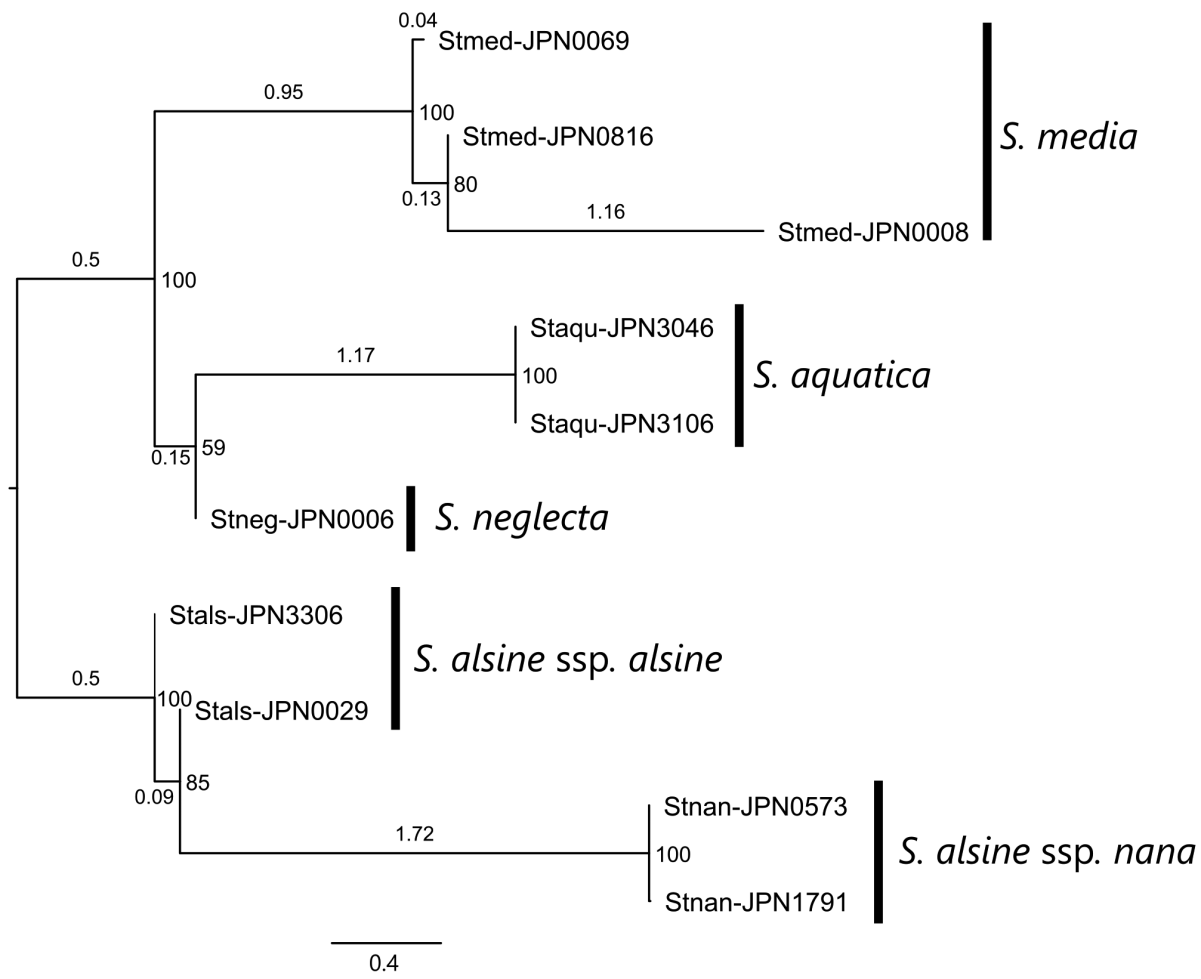
A phylogenetic tree of *Stellariaalsine* and its relatives reconstructed using MIG-seq data. Stals: S.alsinesubsp.alsine. Staqu: *S.aquatica*. Stmed: *S.media*. Stnan: S.alsinesubsp.nana. Stneg: *S.neglecta*. Bootstrap values are shown on the nodes. Branch length represents the average number of substitutions per SNP site.

Fewer shared loci were observed between *S.alsine* and *S.diversiflora*, as well as between *S.alsine* and *S.monosperma*, resulting in the exclusion of *S.diversiflora* and *S.monosperma* from the phylogenetic reconstruction. In the tree obtained (Fig. 1), the sister relationship between S.alsinesubsp.alsine and subsp.nana was supported by a 100% bootstrap value. None of the three species included in the phylogenetic reconstruction, i.e., *S.aquatica*, *S.media*, and *S.neglecta*, are directly related to *S.alsine*.

### ﻿ITS tree

**Figure 2.**
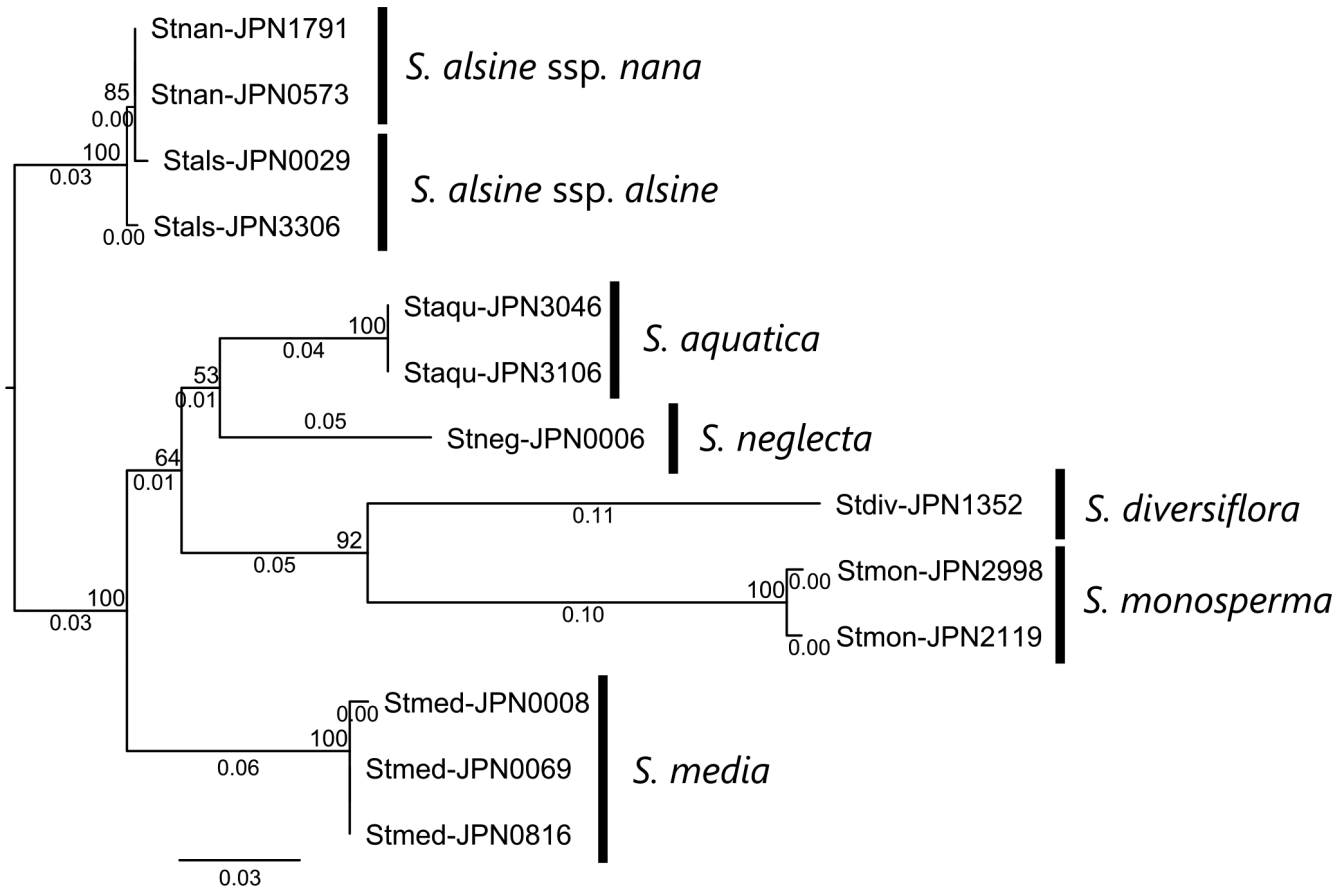
A phylogenetic tree of *Stellariaalsine* and its relatives reconstructed using ITS sequences. Stals: S.alsinesubsp.alsine. Staqu: *S.aquatica*. Stdiv: *S.diversiflora*. Stmed: *S.media*. Stmon: *S.monosperma*. Stnan: S.alsinesubsp.nana. Stneg: *S.neglecta*. Bootstrap values are shown on the nodes. Branch length represents the average number of substitutions per site.

A total of 64,378 reads (4,952 ± 387 reads per sample, ITS1) and 68,362 reads (5,258 ± 357 reads per sample, ITS2) were obtained. After the gaps were trimmed, the total length of the remaining sequences was 662 bp. The monophyly of S.alsinesubsp.alsine and subsp.nana was supported by a 100% bootstrap value (Fig. 2). Moreover, none of the five other species were directly related to *S.alsine*. Additionally, the sister relationship between *S.diversiflora* and *S.monosperma* was supported by a 92% bootstrap value.

### ﻿cpDNA tree

**Figure 3.**
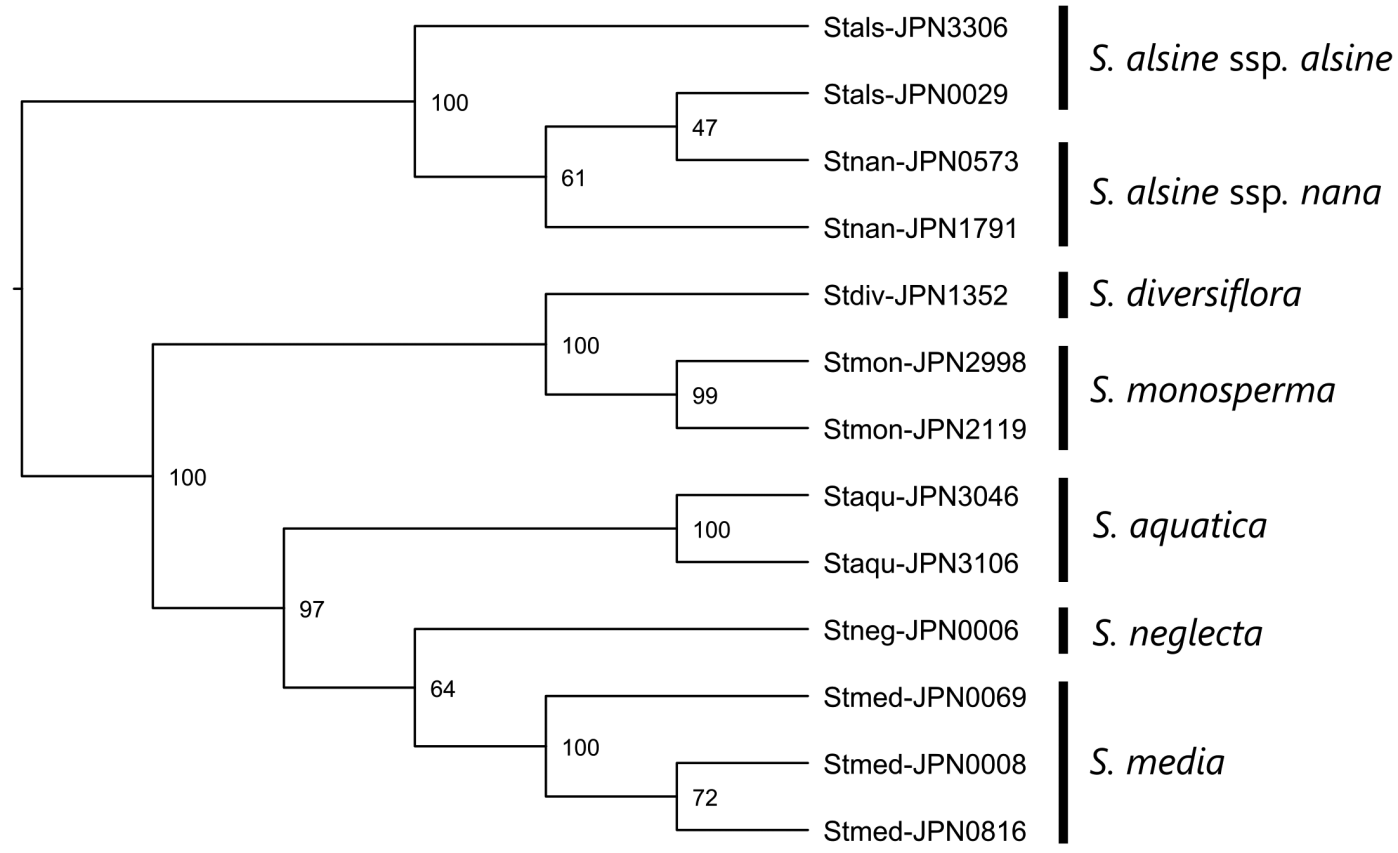
A phylogenetic tree of *Stellariaalsine* and its relatives reconstructed using cpDNA sequences. Bootstrap values are shown on the nodes.

A total of 23,206 reads (1,785 ± 129 reads per sample, *rbc*L), 2,142 reads (165 ± 24 reads per sample, *trn*L intron), and 55,274 reads (4,252 ± 569 reads per sample, *psb*A-*trn*H) were obtained. After the gaps were trimmed, the total length of the remaining sequences was 1,467 bp. The monophyly of S.alsinesubsp.alsine and subsp.nana was supported by a 100% bootstrap value. As observed in the case of ITS, none of the five other species was directly related to *S.alsine* (Fig. 3). Furthermore, the sister relationship between *S.diversiflora* and *S.monosperma* was supported by a 100% bootstrap value.

## ﻿Discussion

Phylogenetic trees obtained using MIG-seq, ITS sequences, and cpDNA sequences supported the monophyly of Stellariaalsinesubsp.alsine and subsp.nana; the bootstrap supports for the monophyly were 100% in all trees. While S.alsinesubsp.nana is a much smaller plant compared to S.alsinesubsp.alsine, both subspecies are similar in their presence of oblong-lanceolate leaves that are glabrous and sessile. Both molecular and morphological evidence supports the deduction that subsp.nana is derived from subsp.alsine by adapting to the mountaintop habitats of Yakushima, where many dwarf endemics are found (Yahara et al. 1987).

In Japan, S.alsinessp.alsine has been identified as S.uliginosaMurrayvar.undulata (Thunb.) Fewnzl. (Kadota 2017), but *S.uliginosa* is generally treated as a synonym of *S.alsine*, which is widely distributed in Europe, Asia, and eastern North America (Chen and Rabeler 2001). A recent molecular phylogenetic study (Sharples 2019) proposed that *S.alsine* is polyphyletic and included two unrelated lineages: one lineage from European Russia belongs to the Nitentes clade, and another one from eastern Asia and eastern North America form the Uliginosae clade. *Stellariaalsine* was described from Europe (Grimm 1767), but Sharples (2019) cited the material from European Russia as ‘S.cf.alsine’, while those from N America and E Asia as ‘*S.alsine*’. In this paper, we followed this treatment for the E Asian lineage as *S.alsines. lat.*

In Japan, S.alsinessp.alsine is a weedy species common in disturbed habitats near farmlands, including paddy fields, and along mountain paths. On the other hand, Stellariaalsinesubsp.nana grows in natural habitats on rocks along streams in the mountainous area of Yakushima, at high elevations of 1500–1700 m. Another example of a weed-derived lineage dwarfed in the mountainous area of Yakushima is PlantagoasiaticaL.var.yakusimensis (Masam.) Ohwi (Plantaginaceae) (Yahara et al. 1987). These examples suggest that dwarfed endemics in Yakushima include lineages not only of ancient origin, such as *Mitelladoiana* Ohwi (Saxifragaceae) (Okuyama et al. 2005), but also those of more recent origin. The discovery of S.alsinesubsp.nana provides new materials for studying the origin and evolution of dwarfed endemic plants in Yakushima.

## ﻿Taxonomy

### ﻿ Stellaria alsine Grimm subsp. nana

Taxon classificationPlantaeCaryophyllalesCaryophyllaceae

K. Fuse & Yahara subsp. nov.

086CE360-D0A8-55C4-AD65-F2EB8867F874

urn:lsid:ipni.org:names:77234845-1

Fig. 4

#### Diagnosis.

Stellariaalsinesubsp.nana differs from the typical subspecies in its shorter stem (2.5–6 cm vs. 15–30 cm), smaller lamina (0.5–1 cm × 1–3 mm vs. 0.5–2 cm × 2–4 mm), shorter pedicels (0.1–0.6 cm long vs. 0.5–2 cm long), as well as chasmogamous flowers with 3 stamens and 2 styles (vs. 5 stamens and 3 styles) that are usually solitary (vs. usually 3–5 on a terminal or axillary cyme).

**Figure 4.**
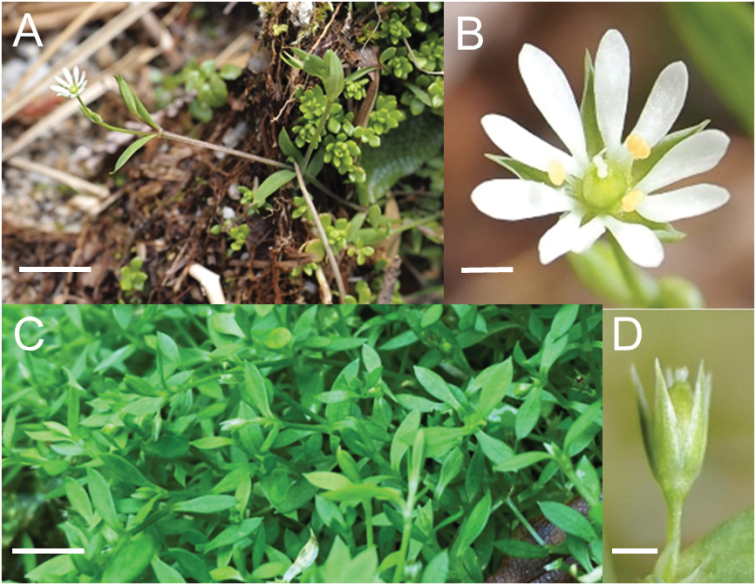
Stellariaalsinesubsp.nana K. Fuse & Yahara **A** a living stem of *K. Fuse & T. Saito JPN4891* bearing a chasmogamous flower **B** the chasmogamous flower of A **C** Living stems of the holotype, *T. Yahara* et al. *JPN*0573, bearing cleistogamous flowers **D** a fruit of *K. Fuse & T. Saito JPN1791.* Scale bars: 1 cm (**A, C**); 1 mm (**B**); 2 mm (**D**).

#### Type.

Japan. Kagoshima Prefecture: Yakushima, along a path to Mt. Nagata, on rocks along streams, 30°20'28.96"N, 130°28'37.22"E, 1500 m elevation, 21 July 2020, with cleistogamous flowers, *T. Yahara*, *H. Sato*, *K. Fuse*, *Y. Higashi JPN0573* (***holotype***: FU!, ***isotype***: KYO!).

#### Description.

Herbs possibly biennial. Stems caespitose, 2.5–6 cm long, glabrous, erect in upper and middle parts, prostrate in lower parts. Leaves deciduous, sessile; blade oblong-obovate or narrowly obovate, 0.5–1 cm × 1–3 mm, glabrous, single-veined, base attenuate, apex acute, margin entire. Flowers solitary or 2–3 flowers in an axillary or terminal cyme with a ca. 6 mm scape. Pedicel 1–6 mm long, slender, glabrous. Sepals ovate-lanceolate, ca. 2.5 mm long in chasmogamous flowers, ca. 2.0 mm long in cleistogamous flowers, glabrous, apex acute. Petals of chasmogamous flowers 5, ca. 2.6 mm long, 2-cleft nearly to base; lobes oblanceolate, apex obtuse; petals absent in cleistogamous flowers. Stamens of chasmogamous flowers 3, 0.8 mm long; filaments ca. 0.4 mm long, glabrous, anthers globular, 0.4 mm in diam. Styles 2, glabrous. Capsule obovoid, 2.5 mm long, as long as sepals when mature, 2-loculated. Seeds dark brown, reniform, ca. 0.7 mm long, slightly flattened, tuberculate with raised papillae, without an appendage.

#### Phenology.

Chasmogamous flowers were observed in May, cleistogamous flowers and immature fruits were observed in July, and mature fruits and seeds were observed in September.

#### Distribution and habitat.

Yakushima, Japan (endemic). At present, this subspecies has been identified in two populations growing on rocks along streams at 1500 m elevation in the vicinity of Mt. Nagata.

#### Etymology.

The subspecific epithet is derived from its dwarf habit.

#### Conservation status.

Vulnerable (VU). The population size was estimated to be between 250 and 1000 mature individuals. The habitat is located within the protected area of Yakushima (Island) National Park and no threats are detected at present.

#### Additional specimens examined.

Japan. Kagoshima Prefecture: Yakushima, along a path to Mt. Nagata, 30°20'28.96"N, 130°28'37.22"E, 1500 m elevation, 4 September 2020, with fruits, *K. Fuse*, *T. Saito JPN1791* (FU!); a gorge N of Mt. Nagata, 30°20'39.7"N, 130°29'28.5"E, 1700 m elevation, 3 May 2021, with chasmogamous flowers, *K. Fuse*, *T. Saito JPN4891* (FU!).

## Supplementary Material

XML Treatment for Stellaria alsine Grimm subsp. nana
